# Comparisons of oncological and functional outcomes among radical retropubic prostatectomy, high dose rate brachytherapy, cryoablation and high-intensity focused ultrasound for localized prostate cancer

**DOI:** 10.1186/s40064-016-3584-4

**Published:** 2016-11-03

**Authors:** Po Hui Chiang, Yi Yang Liu

**Affiliations:** 1 Department of Urology, Kaohsiung Chang Gung Memorial Hospital, No. 123, Dapi Rd, Niaosong District, Kaohsiung City, 833 Taiwan; 2 College of Medicine, Chang Gung University, Taoyuan City, Taiwan

**Keywords:** Brachytherapy, Cryoablation, High-intensity focused ultrasound, Localized prostate cancer, Radical prostatectomy

## Abstract

**Purpose:**

To conduct a retrospective, single institutional and comparative study for radical retropubic prostatectomy (RRP), high dose rate brachytherapy (HDRBT), cryoablation and high-intensity focused ultrasound (HIFU) in localized prostate cancer with respect to oncological and functional outcomes.

**Methods:**

We reviewed 97, 161, 114 and 120 patients of RRP, HDRBT, cryoablation and HIFU respectively for localized prostate cancer from May 2008 to December 2013. PSA biochemical recurrence, salvage treatment-free rate, metastasis-free rate, and biochemical recurrence-free survival were analyzed for oncological outcomes. Functional outcomes included complications and serial IIEF-5 scores, IPSS and related QoL scores.

**Results:**

During nearly 3 years of follow-up, the patients of HDRBT experienced higher PSA biochemical recurrence rate overall (54.7%), as well as D’Amico intermediate-risk (34.4%) and high-risk (61.8%) groups, lower salvage treatment-free rate (46.7%), and metastasis-free rate (90.7%). Besides, the patients of RRP demonstrated higher urethral stricture (29.9%) and urinary incontinence (11.3%). The patients of HIFU revealed lower de novo erectile dysfunction rate at 1 year (65.6%), higher serial IIEF-5 scores, lower IPSS and related QoL scores.

**Conclusions:**

The patients of HDRBT demonstrated worse oncological outcomes in D’Amico intermediate and high-risk groups. Besides, the patients of RRP had more complications rate in urethral stricture and urinary incontinence. Moreover, the patients of HIFU experienced better urinary function improvement and more possible sexual function preservation. In consideration of trifecta, HIFU may provide equivalent cancer control and better quality of life for patients of localized prostate cancer.

## Background

Trifecta, including urinary continence, potency and cancer control, is an important concept for treatment of localized prostate cancer in recent years (Bianco et al. 2005). It not only represents an ideal treatment outcome for localized prostate cancer, but implies the importance of the patients’ quality of life as well. Functional outcome cannot be overemphasized in comparison with oncological outcome and may be the key concern for decision-making of treatment modality, especially for prostate cancer. To date, radical prostatectomy, radiotherapy/brachytherapy, cryoablation, and high-intensity focused ultrasound are used to treat localized prostate cancer (National Comprehensive Cancer N 2014; Mottet et al. 2014). In fact, no single treatment modality has proven superior to the others, and the optimal treatment for localized prostate cancer remains a matter of debate.

To the best of our knowledge, there is a paucity of comparative studies regarding the outcomes of the four treatments; therefore, we conducted a retrospective, single institutional and comparative study evaluating oncological and functional outcomes of the four commonly used treatments for localized prostate cancer.

## Methods

### Patients and study design

This study was approved by the Chang Gung Medical Foundation Institutional Review Board (IRB) for data analysis, and the serial number is 100-1264B. The data were analyzed retrospectively and anonymously. From May 2008 to December 2013, patients with clinically localized prostate cancer (T stage ≦T3a, N0, M0) were reviewed. The clinical stage is decided by Gleason score, digital rectal examination, initial prostate-specific antigen (iPSA), and image studies (bone scan, pelvic computed tomography or magnetic resonance imaging). Among these patients, we excluded the patients undergoing treatments other than the four treatment modalities: (1) Radical retropubic prostatectomy (RRP), (2) High dose rate brachytherapy (HDRBT), (3) Cryoablation and (4) High-intensity focused ultrasound (HIFU). The non-randomized treatment selection was made by the surgeon and patient’s discussion and preference. Finally, there were four treatment groups: (1) RRP (N = 97), (2) HDRBT (N = 161), (3) Cryoablation (N = 114), and (4) HIFU (N = 120).

The perioperative parameters such as age, preoperative prostate volume, iPSA, Gleason score, T stage, D’Amico risk group, the 5-item version of the international index of erectile function (IIEF-5), international prostate symptom score (IPSS) and related quality of life (QoL) score at baseline were collected for demographic data.

Besides, postoperative PSA nadir, time to PSA nadir, PSA biochemical recurrence (for radical retropubic prostatectomy, PSA ≥ 0.2 ng/mL; for the other three treatments, PSA ≥ PSA nadir + 2 ng/mL), salvage treatment-free rate, and metastasis-free rate were checked for oncological outcomes.

In addition, we recorded urethral stricture, second transurethral resection of the prostate (TURP) or optic internal urethrotomy (OIU), urinary incontinence defined one or more daily absorbent pads use, epididymitis, scrotal edema, rectal injury, irradiation cystitis, irradiation proctitis, series of IIEF-5, IPSS and related QoL scores at 6, 12, 18, and 24 months postoperatively for functional outcomes. Postoperative erectile dysfunction was defined by patients with preoperative IIEF-5 ≥ 17 and postoperative IIEF-5 < 17 (moderate to severe erectile dysfunction) at 1 year.

### Treatment techniques

#### Radical retropubic prostatectomy (RRP)

Radical retropubic prostatectomy was performed through a low midline incision and extraperitoneal approach. The extent of radical retropubic prostatectomy included the entire prostate gland, bilateral seminal vesicles and pelvic lymph nodes. Neurovascular bundle sparing technique was not intentionally used.

#### High dose rate brachytherapy (HDRBT)

High dose rate brachytherapy was first performed by urologists in conjunction with radiation oncologists. Iridium radioisotopes (Ir192) were introduced by the radiation oncologists through transperineal needles implanted by urologists in three fractions on two consecutive days. The dose rate was individually designed for each patient on the basis of the results of the examination of important organs such as the urethra, bladder, and rectum, or any defect caused by TURP. The needles were removed after delivering the final HDR fraction. HDR brachytherapy (dose, 4–5.6 Gy per fraction) was performed as a boost followed by external beam radiation therapy (EBRT) (dose, 45–57.6 Gy) 2–3 weeks later. We did not give perioperative androgen deprivation therapy in all the patients (Chiang et al. 2004).

#### Cryoablation

Whole-gland cryoablation was performed by fourth-generation cryosurgical technology (Endocare Cryocare Surgical System, Heathtronics Inc., Austin, TX, USA) under the guidance of transrectal ultrasonography with thermal sensor monitoring, urethral warming, and Denonvilliers’ fascia normal saline instillation to avoid urethral and rectal injury (Liu et al. 2015).

#### High-intensity focused ultrasound (HIFU)

Whole-gland HIFU was performed by Ablatherm^®^ Integrated Imaging (EDAP TMS SA, Vaulx-en-Velin, France) under the guidance of transrectal ultrasonography. In addition, all of the HIFU patients underwent transurethral resection of the prostate (TURP) before operation. If preoperative prostate volume was <30 mL, TURP and HIFU would be performed simultaneously. If preoperative prostate volume was ≥30 mL, HIFU would be performed 4 weeks later after TURP (Liu and Chiang 2016).

### Statistical analysis

All the data was analyzed according to treatment types. Continuous variables were compared by one-way ANOVA test. Categorical variables were compared by Chi square test. Survival analysis was conducted by Kaplan–Meier survival curves and Log Rank test. All analyses were performed using SPSS Statistics version 17.0 (SPSS Inc., Chicago, IL, USA). p values <0.05 were considered significant.

## Results

Patients’ characteristics are listed in Table 1. Preoperative mean IIEF-5, IPSS and QoL scores were similar among the four groups. Nevertheless, other characteristics were different with statistical significance, indicating the different compositions.

**Table 1:** Patients’ characteristics

Variable	RRP (N = 97)	HDR (N = 161)	Cryo (N = 114)	HIFU (N = 120)	P value
Age, years (mean ± SD)	63.53 ± 6.71	71.92 ± 7.03	69.76 ± 6.49	68.06 ± 1.91	0.000
Preoperative prostate volume, mL (mean ± SD)	37.71 ± 16.97	37.48 ± 18.58	36.71 ± 16.94	21.97 ± 10.90	0.000
iPSA, ng/mL (mean ± SD)	16.08 ± 23.38	23.34 ± 20.40	26.76 ± 49.33	17.04 ± 21.88	0.002
iPSA ≤ 10 ng/mL, N (%)	40 (41.2)	39 (24.2)	39 (34.2)	54 (45.0)	0.000
iPSA 10–20 ng/mL, N (%)	37 (38.1)	46 (28.6)	39 (34.2)	38 (31.7)	
iPSA ≥ 20 ng/mL, N (%)	20 (20.6)	76 (47.2)	36 (31.6)	28 (23.3)	
Gleason score					0.002
≤6, N (%)	38 (39.2)	82 (50.9)	41 (36.0)	36 (30.0)	
7, N (%)	41 (42.3)	45 (28.0)	38 (33.3)	57 (47.5)	
≥8, N (%)	18 (18.6)	34 (21.1)	35 (30.7)	27 (22.5)	
T stage					0.000
<T2b, N (%)	16 (16.5)	26 (16.1)	52 (45.6)	73 (60.8)	
T2b, N (%)	7 (7.2)	34 (21.1)	16 (14.0)	14 (11.7)	
>T2b, N (%)	74 (76.3)	101 (62.7)	46 (40.4)	33 (27.5)	
D’Amico risk group					0.000
Low, N (%)	9 (9.3)	6 (3.7)	19 (16.7)	15 (12.5)	
Intermediate, N (%)	10 (10.3)	32 (20.0)	24 (21.1)	47 (39.2)	
High, N (%)	78 (80.4)	123 (76.4)	71 (62.3)	58 (48.3)	
IIEF-5 ≥ 17, preoperative, N (%)	38 (39.2)	34 (21.1)	50 (43.9)	32 (26.7)	0.000
IIEF-5, preoperative^a^ (mean ± SD)	23.61 ± 2.13	22.88 ± 1.81	22.96 ± 2.44	22.10 ± 2.62	0.054
IPSS, preoperative (mean ± SD)	11.64 ± 8.84	8.51 ± 7.73	11.73 ± 7.53	10.16 ± 7.24	0.100
QoL, preoperative (mean ± SD)	3.60 ± 1.96	3.06 ± 2.01	3.11 ± 1.74	2.91 ± 1.69	0.266

*RRP* radical retropubic prostatectomy, *HDR* high dose rate brachytherapy, *Cryo* cryoablation, *HIFU* high-intensity focused ultrasound, *SD* standard deviation, *iPSA* initial prostate-specific antigen, *IIEF-5* 5-item version of the international index of erectile function, *IPSS* international prostate symptom score, *QoL* quality of life

^a^Mean scores for IIEF-5 were restricted to those men who reported IIEF-5 ≥ 17 at baseline

**Fig. 1:**
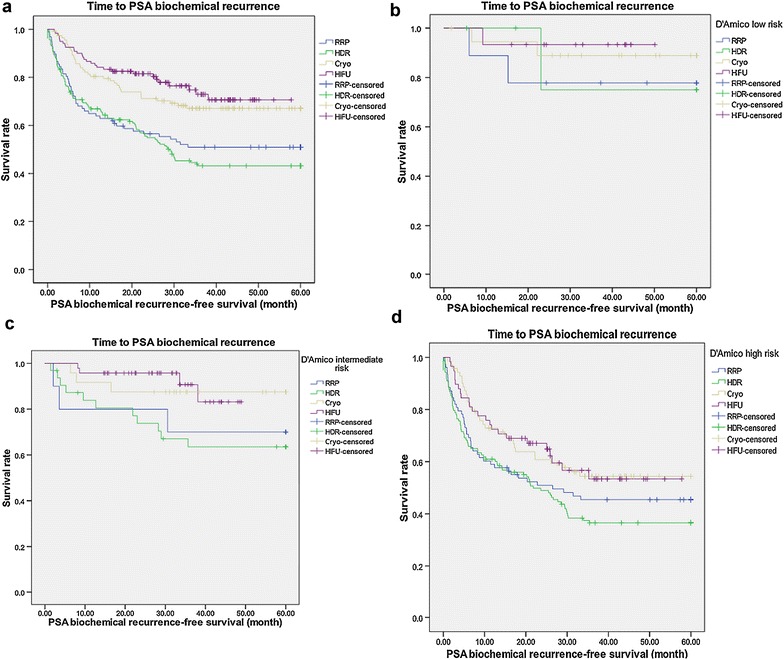
Kaplan–Meier analysis for PSA biochemical recurrence-free survival among primary radical retropubic prostatectomy, high dose rate brachytherapy, cryoablation and high-intensity focused ultrasound **a** overall **b** D’Amico low risk **c** D’Amico intermediate risk **d** D’Amico high risk; RRP radical retropubic prostatectomy, *HDR* high dose rate brachytherapy, *Cryo* cryoablation, *HIFU* high-intensity focused ultrasound, *PSA* prostate-specific antigen

Intergroup oncological outcome comparison is demonstrated in Table 2. During similar mean follow-up duration of nearly 3 years, the patients of HDRBT demonstrated higher PSA biochemical recurrence rate overall (54.7%), as well as D’Amico intermediate-risk (34.4%) and D’Amico high-risk (61.8%) groups than did patients of the other three treatments with statistical significance. Kaplan–Meier analysis for biochemical recurrence-free survival also revealed compatible results (Fig. 1). (Log Rank test: overall P = 0.000, D’Amico low-risk P = 0.689, intermediate-risk P = 0.043, high-risk P = 0.027). Moreover, the patients of HDRBT had significantly lower salvage treatment-free rate (46.7%) and metastasis-free rate (90.7%) than did patients of the other three treatments.

**Table 2:** Oncological outcome comparison

Variable	RRP (N = 97)	HDR (N = 161)	Cryo (N = 114)	HIFU (N = 120)	P value
Follow-up duration, months (mean ± SD)	34.68 ± 12.45	33.54 ± 14.95	33.46 ± 14.38	32.68 ± 11.87	0.338
PSA nadir, ng/mL (mean ± SD)	0.40 ± 1.10	0.94 ± 1.59	0.81 ± 2.29	0.64 ± 1.77	0.031
PSA biochemical recurrence, N (%)	47 (48.5)	88 (54.7)	36 (31.6)	29 (24.2)	0.000
Low risk, N (%)	2 (22.2)	1 (16.7)	2 (10.5)	1 (6.7)	0.699
Intermediate risk, N (%)	3 (30.0)	11 (34.4)	3 (12.5)	4 (8.5)	0.020
High risk, N (%)	42 (53.8)	76 (61.8)	31 (43.7)	24 (41.4)	0.024
PSA biochemical recurrence-free survival, months (mean ± SD)	22.13 ± 14.85	21.17 ± 14.49	26.39 ± 12.53	27.66 ± 13.72	0.000
Salvage treatment free, N (%)	59 (60.8)	75 (46.7)	82 (71.9)	84 (70.0)	0.000
Metastasis free, N (%)	92 (94.8)	146 (90.7)	113 (99.1)	119 (99.2)	0.001

*RRP* radical retropubic prostatectomy, *HDR* high dose rate brachytherapy, *Cryo* cryoablation, *HIFU* high-intensity focused ultrasound, *SD* standard deviation, *PSA* prostate-specific antigen

Intergroup functional outcome comparison is reported in Table 3. In terms of postoperative complications, the patients of RRP had significantly higher rate of urethral stricture (29.9%), secondary TURP or OIU (28.9%), urinary incontinence (11.3%) than did those of the other three treatments. Irradiation cystitis (3.7%) and irradiation proctitis (4.3%) were unique for the patients of HDRBT. Similarly, transient scrotal edema (74.7%) was common for the patients of cryoablation. Rectal injury was only seen in the patients of RRP (1.0%) and HDRBT (1.9%).

**Table 3:** Functional outcome comparison

Variable	RRP (N = 97)	HDR (N = 161)	Cryo (N = 114)	HIFU (N = 120)	P value
Urethral stricture, N (%)	29 (29.9)	10 (6.2)	4 (3.3)	13 (10.8)	0.000
Secondary TURP or OIU, N (%)	28 (28.9)	18 (11.2)	10 (8.8)	16 (13.3)	0.000
Urinary incontinence, N (%)	11 (11.3)	1 (0.6)	2 (1.6)	3 (2.5)	0.000
Epididymitis, N (%)	2 (2.1)	6 (3.7)	8 (7.3)	7 (5.8)	0.311
Scrotal edema, N (%)	0 (0.0)	0 (0.0)	85 (74.7)	0 (0.0)	0.000
Rectal injury, N (%)	1 (1.0)	3 (1.9)	0 (0.0)	0 (0.0)	0.243
Irradiation cystitis, N (%)	0 (0.0)	6 (3.7)	0 (0.0)	0 (0.0)	0.006
Irradiation proctitis, N (%)	0 (0.0)	7 (4.3)	0 (0.0)	0 (0.0)	0.002
Erectile dysfunction at 12 months, N (%)^a^	32/38 (84.2)	30/34 (88.2)	44/50 (88.0)	21/32 (65.6)	0.042
IIEF-5 at 6 months (mean ± SD)^a^	6.08 ± 6.27	4.85 ± 5.52	4.02 ± 5.95	8.55 ± 8.41	0.031
IIEF-5 at 12 months (mean ± SD)^a^	6.33 ± 6.06	4.81 ± 5.47	3.61 ± 5.21	9.67 ± 7.74	0.003
IIEF-5 at 18 months (mean ± SD)^a^	6.74 ± 6.07	4.79 ± 5.32	4.50 ± 5.96	10.16 ± 8.11	0.008
IIEF-5 at 24 months (mean ± SD)^a^	5.48 ± 5.28	4.76 ± 5.48	4.18 ± 5.89	9.36 ± 6.33	0.067
IPSS at 6 months (mean ± SD)	9.55 ± 6.11	7.51 ± 5.81	10.43 ± 6.50	7.26 ± 4.41	0.009
IPSS at 12 months (mean ± SD)	9.69 ± 6.01	7.48 ± 5.56	9.54 ± 5.87	6.25 ± 3.42	0.021
IPSS at 18 months (mean ± SD)	9.52 ± 6.05	7.49 ± 5.67	9.15 ± 6.08	5.82 ± 3.75	0.047
IPSS at 24 months (mean ± SD)	9.31 ± 5.83	7.50 ± 5.43	9.04 ± 6.30	5.70 ± 3.53	0.184
QoL at 6 months (mean ± SD)	2.50 ± 1.59	2.27 ± 1.33	2.75 ± 1.41	1.90 ± 0.14	0.017
QoL at 12 months (mean ± SD)	2.52 ± 1.57	2.30 ± 1.42	2.59 ± 1.42	1.83 ± 1.01	0.097
QoL at 18 months (mean ± SD)	2.45 ± 1.56	2.32 ± 1.28	2.5 ± 1.41	1.86 ± 1.17	0.318
QoL at 24 months (mean ± SD)	2.50 ± 1.65	2.33 ± 1.47	2.47 ± 1.40	1.98 ± 1.4	0.794

*RRP* radical retropubic prostatectomy, *HDR* high dose rate brachytherapy, *Cryo* cryoablation, *HIFU* high-intensity focused ultrasound, *TURP* transurethral resection of the prostate, *OIU* optic internal urethrotomy, *SD* standard deviation, *IIEF-5* 5-item version of the international index of erectile function, *IPSS* international prostate symptom score, *QoL* quality of life

^a^Erectile dysfunction and Mean scores for IIEF-5 were restricted to those men who reported IIEF-5 ≥ 17 at baseline

**Fig. 2:**
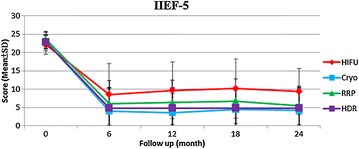
Mean scores for IIEF-5 among primary radical retropubic prostatectomy, high dose rate brachytherapy, cryoablation and high-intensity focused ultrasound; *RRP* radical retropubic prostatectomy, *HDR* high dose rate brachytherapy, *Cryo* cryoablation, *HIFU* high-intensity focused ultrasound, *IIEF-5* 5-item version of the international index of erectile function; note: mean scores for IIEF-5 were restricted to those men who reported IIEF-5 ≥ 17 at baseline

There were 38, 34, 50 and 32 patients of RRP, HDRBT, cryoablation and HIFU whose IIEF-5 score was ≥17 at baseline respectively. The mean preoperative IIEF-5 score of these patients was similar. The four groups of patients had a decline in IIEF-5 score postoperatively. However, the patients of HIFU experienced significantly lower postoperative erectile dysfunction rate at 12 months (65.6%, P = 0.042) and higher serial IIEF-5 score at 6, 12 and 18 months than those of the other three treatments (Fig. 2).

**Fig. 3:**
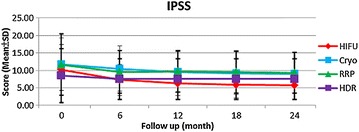
Mean scores for IPSS among primary radical retropubic prostatectomy, high dose rate brachytherapy, cryoablation and high-intensity focused ultrasound; *RRP* radical retropubic prostatectomy, *HDR* high dose rate brachytherapy, *Cryo* cryoablation, *HIFU* high-intensity focused ultrasound, *IPSS* international prostate symptom score

**Fig. 4:**
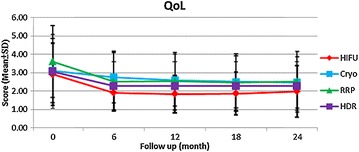
Mean scores for QoL among primary radical retropubic prostatectomy, high dose rate brachytherapy, cryoablation and high-intensity focused ultrasound; *RRP* radical retropubic prostatectomy, *HDR* high dose rate brachytherapy, *Cryo* cryoablation, *HIFU* high-intensity focused ultrasound, *QoL* quality of life

The four groups of patients had similar preoperative IPSS and QoL scores. Postoperatively, all the four groups experienced decreased IPSS and QoL scores from baseline, indicating improvement of urinary function. Postoperatively, the patients of HIFU had significantly lower IPSS at 6, 12, and 18 months and lower QoL scores at 6 and 12 months than did those of the other three treatments (Figs. 3, 4).

## Discussion

The dilemma between definitive treatment and observation/watchful waiting for localized prostate cancer is an important question without standard answer. In the PIVOT study, radical prostatectomy did not significantly reduce all-cause or prostate-cancer specific mortality, as compared with observation during at least 12 years of follow-up (Wilt et al. 2012). In contrast, the SPCG-4 study reported that radical prostatectomy significantly reduced all cause mortality by 12.7% (P < 0.001) and prostate-cancer specific mortality by 11.0% (P = 0.001) compared to watchful waiting during 23.2 years of follow-up (Bill-Axelson et al. 2014). Besides, surgical treatment for localized prostate cancer will inevitably result in complications and functional impairments. Therefore, an ideal treatment for localized prostate cancer with acceptable cancer control and less complications can avoid not only overtreatment and negative impact on quality of life but also disease progression and anxiety related to observation/watchful waiting.

With regard to oncological outcome comparisons, the patients of HDRBT experienced the worst cancer control in PSA biochemical recurrence (overall, D’Amico intermediate and high risk), salvage treatment-free rate and metastasis-free rate compared to those of the other three treatments. Even the patient distribution by D’Amico risk group is not equal in the four treatment modalities statistically, this result still reflects the insufficiency of pure HDRBT without androgen deprivation therapy for D’Amico intermediate and high-risk patients. According to NCCN guidelines and previous studies, neoadjuvant/concomitant/adjuvant androgen deprivation therapy is suggested for intermediate-risk (4–6 months) and high-risk patients (2–3 years) (National Comprehensive Cancer N 2014; Zumsteg et al. 2013; Bolla et al. 2010; D’Amico et al. 2008; Bolla et al. 2002). Our results are compatible with this suggestion. In the present study, cryoablation and HIFU demonstrated non-inferior oncologic outcomes to RRP, which is considered as standard treatment for localized prostate cancer. This intermediate-term result appears to be promising and needs further follow-up.

In respect of postoperative complications, RRP resulted in more urinary incontinence (11.3%), urethral stricture (29.9%) and secondary TURP or OIU (28.9%) than did the other three treatments. In the literature review, post-RRP urinary incontinence rate decreased with time (49.5% at 3 months, 9.5% at 24 months). Finally, 1–3% patients needed surgical intervention (Namiki and Arai 2010). Even in the era of robotic surgery, we still cannot find significant improvement of urinary incontinence rate after robotic-assisted laparoscopic radical prostatectomy compared with open radical prostatectomy (Haglind et al. 2015). Although we may expect the possibly partial recovery of urinary incontinence after a long time, post-RRP urinary incontinence is still a major concern for patients’ quality of life, especially for younger patients with longer life expectancy.

In terms of sexual function, the patients of HIFU demonstrated significantly lower de novo erectile dysfunction rate at 12 months (65.5%) and higher serial IIEF-5 scores than did those of the other three treatments, indicating more possibility of sexual function preservation. In a prospective study comparing radical prostatectomy, brachytherapy and cryoablation, brachytherapy led to better sexual function and bother scores than did radical prostatectomy and cryoablation for 3 years (Malcolm et al. 2010). We did not see the trend in this study, probably due to significantly older age in the patients of HDRBT. Another prospective study revealed that patients of HIFU had continuously better postoperative IIEF scores than cryoablation for 3 years (Li et al. 2010). Our results were compatible with this finding. It is worth noting that the real serial IIEF-5 scores of RRP may be underestimated because the neurovascular bundle sparing technique was not intentionally used in this study. In a recent systemic review, mean potency recovery rates at 12 months can reach 55–81% for patients treated with robotic-assisted laparoscopic prostatectomy and 26–63% for patients treated with retropubic radical prostatectomy (Ficarra et al. 2012). Thus, the potency rate may increase with the improvement of surgical device and technique. Besides, the patients of HIFU experienced significantly lower serial IPSS and QoL scores than did those of the other three treatments, implying better urinary function improvement. The previous study comparing radical prostatectomy, brachytherapy and cryoablation reported that the patients of brachytherapy and cryotherapy had better urinary function and bother scores than those undergoing radical prostatectomy (Malcolm et al. 2010). These conclusions were similar to our results. To sum up, the outcomes for sexual and urinary function are convincing and highlight that HIFU may be an alternative choice than the other three treatments with regard to quality of life.

In our experience, the delicate surgical margin is more easily to be controlled by HIFU because of the computer-programmed targeted area by 0.06 mL each time. Therefore, the precise “nerve-sparing” or focal HIFU decreases the erectile dysfunction rate to 22–31% and results in better sexual function preservation (Shoji et al. 2010; Poissonnier et al. 2007). Moreover, we routinely performed TURP before HIFU. The advantages of TURP before HIFU are (1) to reduce the prostate volume, especially ventral, apical or intravesical prostate tissue to avoid incomplete treatment; (2) to remove prostatic calcification or abscess that would attenuate the HIFU energy; and (3) to reduce the postoperative obstruction complication rate (from 31 to 6%) (Poissonnier et al. 2007; Chaussy and Thuroff 2003; Netsch et al. 2010). In other words, TURP before HIFU is the key point for successful oncological and functional outcomes.

Some limitations of this study should be noted. First, it was a retrospective and non-randomized study. Therefore, the inclusion criteria of the four treatment modalities cannot be defined clearly and the baseline data for the four groups cannot be totally equal. In fact, it is difficult to conduct a randomized controlled trial for surgical devices in the real world. It is a matter of patients’ performance, preference, medical cost and clinicians’ experience. Though the patients’ characteristics have statistically significant differences in each group, we compare the PSA biochemical recurrence rate in D’Amico low, intermediate and high risk group respectively, according to initial PSA, clinical stage and Gleason score. This kind of comparison may be more objective and alleviate the patient selection bias. In addition, the mean follow-up duration of nearly 3 years was relatively shorter for oncological outcomes. However, it may be sufficient for evaluation of the functional outcomes in accordance with previous studies. Finally, postoperative sexual function of patients undergoing RRP should be carefully interpreted due to lack of neurovascular bundle sparing technique in this study.

To the best of our knowledge, it is the first single institutional and comparative study discussing comparison of the oncological and functional outcomes of radical retropubic prostatectomy, high dose rate brachytherapy, cryoablation and high-intensity focused ultrasound for localized prostate cancer. Besides, there is no inter-institutional and inter-operator bias in this study because except for radiation oncologists, almost all patients undergoing these four treatments (radical retropubic prostatectomy, high dose rate brachytherapy, cryoablation and HIFU) had them performed by the same surgeon (Dr. Po Hui Chiang). Moreover, this study will provide useful information and give rise to the interest of urological oncologists. We look forward to further prospective, randomized controlled trials in the treatment of localized prostate cancer in the future.

## Conclusion

Among the four treatments, the patients of high dose rate brachytherapy demonstrated the worst oncological outcomes, especially in D’Amico intermediate- and high-risk groups. Besides, the patients of radical retropubic prostatectomy had more complications rate in urethral stricture and urinary incontinence. Moreover, the patients of HIFU experienced better urinary function improvement and more possible sexual function preservation. In consideration of trifecta, HIFU may provide equivalent cancer control in the intermediate-term follow-up and better quality of life for patients of localized prostate cancer.
